# Outdoor air pollution, green space, and cancer incidence in Saxony: a semi-individual cohort study

**DOI:** 10.1186/s12889-018-5615-2

**Published:** 2018-06-08

**Authors:** Thomas Datzmann, Iana Markevych, Freya Trautmann, Joachim Heinrich, Jochen Schmitt, Falko Tesch

**Affiliations:** 1 TU Dresden, Medizinische Fakultät Carl Gustav Carus, Center for Evidence-Based Healthcare, Dresden, Germany; 2 National Center for Tumor Diseases, Dresden, Germany; 3 LMU Munich, University Hospital, Institute and Outpatient Clinic for Occupational, Environmental and Social Medicine, Munich, Germany; 4 Helmholtz Zentrum München, German Research Center for Environmental Health, Institute of Epidemiology I, Neuherberg, Germany

**Keywords:** Cancer incidence, Air pollution, Particulate matter, Nitrogen dioxide, Green space

## Abstract

**Background:**

There are a few epidemiological studies that (1) link increased ambient air pollution (AP) with an increase in lung cancer incidence rates and (2) investigate whether residing in green spaces could be protective against cancer. However, it is completely unclear whether other forms of cancer are also affected by AP and if residential green spaces could lower cancer incidence rates in general. Therefore, the objective was to estimate whether AP and green space are associated with several cancer types.

**Methods:**

The analysis was based on routine health care data from around 1.9 million people from Saxony who were free of cancer in 2008 and 2009. Incident cancer cases (2010–2014) of mouth and throat, skin (non-melanoma skin cancer - NMSC), prostate, breast, and colorectum were defined as: (1) one inpatient diagnosis, or (2) two outpatient diagnoses in two different quarters within one year and a specific treatment or death within two quarters after the diagnosis. Exposures, derived from freely available 3rd party data, included particulate matter with aerodynamic diameter of less than 10 μm (PM_10_) and nitrogen dioxide (N0_2_) as well as green space (Normalized Difference Vegetation Index - NDVI). Associations between air pollutants, green space, and cancer incidence were assessed by multilevel Poisson models. Age, sex, physician contacts, short- and long-term unemployment, population density, and having an alcohol-related disorder were considered as potential confounders.

**Results:**

Three thousand one hundred seven people developed mouth and throat cancer, 33,178 NMSC, 9611 prostate cancer, 9577 breast cancer, and 11,975 colorectal cancer during the follow-up period (2010–2014). An increase in PM_10_ of 10 μg/m^3^ was associated with a 53% increase in relative risk (RR) of mouth and throat cancer and a 52% increase in RR of NMSC. Prostate and breast cancer were modestly associated with PM_10_ with an increase in RR of 23 and 19%, respectively. The associations with N0_2_ were in the same direction as PM_10_ but the effect estimates were much lower (7–24%). A 10% increase in NDVI was most protective of mouth and throat cancer (− 11% RR) and of NMSC (− 16% RR). Colorectal cancer was not affected by any of the exposures.

**Conclusions:**

In addition to the studies carried out so far, this study was able to provide evidence that higher ambient AP levels increase the risk of mouth and throat cancer as well as of NMSC and that a higher residential green space level might have a protective effect for NMSC in areas with low to moderate UV intensity. Nevertheless, we cannot rule out residual confounding by socioeconomic or smoking status.

**Electronic supplementary material:**

The online version of this article (10.1186/s12889-018-5615-2) contains supplementary material, which is available to authorized users.

## Background

Outdoor air pollution (AP) is ubiquitous with its exposure having effects on a large proportion of the world population [1]. There is strong evidence from experimental and epidemiological studies that AP such as particulate matter (PM), nitrogen dioxide (NO_2_), and ozone (O_3_) are major risk factors for cardiovascular and cardiopulmonary diseases, and potentially cancer [2–9]. While the trigger function of AP on cardiac and pulmonary events is considered as causal, the role in cancer onset is only suggestive. In the case of PM and cardiovascular mortality, there is a solid association that fulfils both a temporal and a close exposure-response relationship. There is coherence of results between several scientific disciplines, including experimental studies offering plausible biological mechanisms reviewed in [3]. Both long-term and short-term effects are involved. An increase in PM_2.5_ long-term exposure per 10 μg/m^3^ increased the risk for cardiovascular mortality by 11% in a recent meta-analysis [5]. Even a daily rise in mean PM_2.5_ level per 10 μg/m^3^ increased the risk for cardiovascular mortality by approximately 0.4 to 1.0% [10]. In the case of cancer, it is much more difficult to prove causality. One reason is the long latency between exposure to carcinogens and the development of cancer. Another reason is that doses are typically low in the environment, and therefore direct causal inferences are hardly realized. Nevertheless, there is suggestive epidemiological evidence that outdoor AP increases incidence rates of some cancer types with strongest evidence for lung cancer [11–19]. Molecular epidemiological studies [20–22] have shown that the biological mechanisms causing cancer from outdoor AP involve genotoxic effects of the chemical compounds that accumulate over time, including PAH-DNA adducts, chromosome aberrations, sister chromatid exchanges, *ras* oncogene overexpression, and radically induced (oxidative) DNA damage. PM is listed as Group I carcinogen by the International Agency for Research on Cancer (IARC). Sources of PM, other than household, are numerous including traffic, agricultural and industrial emissions as main anthropogenic origins [23, 24]. We use PM_10_ (particles with aerodynamic diameter of less than 10 μm) instead of PM_2.5_, because we have more variation in PM_10_ data. PM_2.5_ is far more evenly distributed throughout Saxony. We use NO_2_as marker for traffic-related AP to investigate the effect of urban agglomeration in Saxony on cancer incidence [25, 26]. We also want to narrow the research gap on the impact of green spaces on health, especially cancer. While numerous studies have analyzed associations between AP and lung cancer, few studies have addressed associations between green space and cancer incidence [27–30]. Green space is known to be inversely related to AP due to lack of emission sources in green places [31]. Additionally, people residing in greener places might be more motivated to conduct physical activity [31], which is suggested to decrease cancer risk [32] through reductions of adipose tissue volume and endocrine activity (e.g. sex hormones) [33, 34]. On the other hand, some studies have also reported adverse associations with green space that may be context-specific, like a negative effect on the human skin through increased sunlight exposure, while spending time in green space [31]. Since a handful of studies have reported a link between lower all-cause mortality among people residing in green spaces [28, 35–37], it is a valid assumption that residential green space might decrease risk of some cancer types.

The aim of the study was to analyze the association between residential exposure to PM_10_ and NO_2_ as well as green space on different cancer types. We restricted the analysis to those cancer types, which are less strongly associated with smoking as lung cancer is, but which might be affected by AP exposure either by inhalation or dermal route of exposure to PM_10_ particles and NO_2_. Further, we needed a sufficient number of cases to ensure that we have enough power for our analyses. Therefore, we had to use cancers that are frequent in the population. We identified mouth and throat cancer, non-melanoma skin cancer (NMSC) and colorectal cancer in both sexes, as well as prostate cancer in men and breast cancer in women, which fulfilled these requirements. At the beginning of the study, we also wanted to examine the effects of pesticides on various forms of cancer in addition to AP and green spaces. According to the literature [15, 16], glandular tissues like the prostate or the breast may be susceptible to some agricultural used pesticides (hormone disruptive agents). Unfortunately, the data situation was not sufficient and therefore this investigation was not continued. Nevertheless, prostate and breast cancer were still used as outcomes.

## Methods

### AOK PLUS study population and case definitions

In Germany, approximately 90% of the population is covered by statutory health insurances. We used routine health care data from AOK PLUS, a large statutory health insurance in Saxony (area ~ 18,000 km^2^, population ~ 4 Mio), which covers almost half of the local general population. The data include information from inpatient and outpatient care with respect to diagnosis, procedures and prescriptions as well as socio-demographic information of the insured population such as age, sex and residential district (first four digits of the 5-digit postal code of the residential address). Age distribution and sex-ratio of the AOK PLUS beneficiaries in Saxony are comparable to the Germany-wide population [38]. We used data for the years 2007–2014 from the outpatient as well as from the inpatient sector. All beneficiaries were allocated to 186 four digit postal code districts based on their residential address in 2007. We excluded all cancer cases from the year 2007 for the determination of prevalent cases, because we had only outpatient data for this year. Further, beneficiaries that were diagnosed (outpatient or inpatient) in the years 2008 and 2009 with one of the analyzed cancer types were excluded, to estimate incidence rates for the years 2010 until 2014 (see Additional file 1: Figure S3). Age groups in increments of 10 years were built based on the age of every beneficiary in the year 2012. Therefore, the newborns of the years 2013 and 2014 had to be excluded as well. Further, the ages from 0 to 49 years were collapsed to one group. See Table 1 for an exact partitioning of ages.

**Table 1:** Baseline characteristics of the study population (AOK PLUS data)

Characteristics of study population	Insured (total)		Colorectal cancer		Mouth and throat cancer		NMSC		Prostate cancer		Breast cancer	
	n	%	n	%	n	%	n	%	n	%	n	%
Total (2010–2014)	1,918,449	100	11,976	100	3107	100	33,178	100	9611	100	9577	100
Sex												
Male	897,417	46.78	6295	52.56	2305	74.19	16,680	50.27	9611	100	0	0.00
Female	1,021,032	53.22	5681	47.44	802	25.81	16,498	49.73	0	0.00	9577	100
Age in 2012												
0–49 years	909,067	47.39	307	2.56	320	10.30	1006	3.03	57	0.59	1024	10.69
50–59 years	272,036	14.18	987	8.24	858	27.62	2130	6.42	670	6.97	1585	16.55
60–69 years	220,576	11.50	1861	15.54	716	23.04	5183	15.62	2071	21.55	1893	19.77
70–79 years	269,933	14.07	4161	34.74	736	23.69	13,069	39.39	4339	45.15	2617	27.33
80–89 years	190,653	9.94	3746	31.28	400	12.87	9704	29.25	2128	22.14	2032	21.22
90+ years	56,184	2.93	914	7.63	77	2.48	2086	6.29	346	3.60	426	4.45
Mean age in 2012 (SD)	49.33 (25.33)		75.04 (11.48)		65.11 (12.99)		74.78 (11.14)		73.68 (9.12)		68.82 (14.08)	
Alcohol related disorder												
yes	69,722	3.63	608	5.08	1118	35.98	844	2.54	538	5.60	197	2.06
no	1,848,727	96.37	11,368	94.92	1989	64.02	32,334	97.46	9073	94.40	9380	97.94
Changed place of residence between 2007 and 2014												
yes	302,818	15.78	1118	9.34	326	10.49	2450	7.38	640	6.66	888	9.27
no	1,615,631	84.22	10,858	90.66	2781	89.51	30,728	92.62	8971	93.34	8689	90.73

NMSC – non-melanoma skin cancer

In accordance with the Good epidemiological practice for secondary data analyses [39], cancer diseases were defined as one diagnosis of the corresponding ICD-10 code (International Classification of Diseases) for the inpatient data, while two diagnoses in different quarters within one year were necessary for all outpatient data plus a prescription of a specific treatment (e.g. radiotherapy, cytostatic medication) or death within two quarters after the second diagnosis (see Additional file 2: Table S1, Additional file 3: Table S2, Additional file 4: Table S3). We used the codes C00-C14 for mouth and throat cancer, C44 or L57.0 for NMSC, C61 for prostate cancer, C50 for breast cancer, and C18-C21 for colorectal cancer.

### Ethics approval and consent to participate

The present analysis is based on secondary data from the health insurance company AOK PLUS, which were collected for the purpose of billing medical services. The study is supported by the AOK PLUS with which a data use and transfer agreement exists. The data was available for us only in anonymous form, so that no conclusions could be drawn about the individuals. Personal data of participants were anonymized through AOK PLUS before data sharing. Personal identifiers were masked or deleted (clear name to pseudonym; no social security number provided). Quasi-identifiers were generalized (only year of birth used; dropping of last digit of the zip code). Unfortunately, the data are not publicly accessible. There was no influence whatsoever on the policyholders and no intervention was carried out. It is therefore a purely observational study. According to paragraph 75 SGB X (Zehntes Buch Sozialgesetzbuch - German federal law) it is not reasonable to get consent for data sharing and analysis from around 2 million people, like in our investigation. Therefore, we submitted and get granted an application to the Saxon State Ministry for Social Affairs and Consumer Protection for obtaining consent to data transmission and analysis on behalf of the insured.

This study was conducted in accordance with the Helsinki Declaration [40] and follows the principles of Good Epidemiological Practice and Good Practice in Secondary Data Analysis [41]. The study was also registered in the database *“Versorgungsforschung Deutschland”* under the number VfD_ECo_epi_16_003770.

### Air pollution and green space exposure assessment

Annual NO_2_ and PM_10_ concentrations for the year 2007 were derived from freely available maps in resolution of 100 m developed for Western Europe [42]. These maps were created by land use regression models based on more than 1500 EuroAirnet monitoring sites. Predictor variables for land use regression models included land use characteristics, population density, road length, altitude, distance to sea, and satellite-derived NO_2_ and PM_10_ data [43].

Green spaces were defined by the Normalized Difference Vegetation Index (NDVI), which was derived from freely available MODerate-resolution Imaging Spectroradiometer (MODIS) satellite images at the resolution of 250 m [44]. Briefly, NDVI is a commonly used indicator of vegetation level, ranging from − 1 (water) to + 1 (absolutely vegetated area). The algorithm for NDVI is based on two vegetation-informative bands: near-infrared (841 nm to 876 nm) and visible red (from 620 nm to 670 nm). For current analysis, we averaged 115 16-day composite NDVI images for the years 2005 to 2009 [45, 46].

These PM_10_, NO_2_, and NDVI estimates for 386 five digit postal codes were weighted by the population number from the German census 2011 and then averaged to 186 four digit postal code districts, because the address information were only available for the four digit postal codes due to privacy regulations. Freely available postal code vector data were obtained from postleitzahl.org.

Geographic data management and calculations were conducted using the ArcGIS 10.1 Geographical Information System (GIS) (ESRI, Redlands, CA, USA) software program.

### Confounders/effect modifiers

As control variables for the individual data analysis, age, sex, alcohol-related disorder, absolute number of physician contacts in the four digit postal code districts (AOK data) and the proportion of short- (up to one year) and long-term unemployment (more than 1 year; statistical office Saxony), ranging from 0 to 1, were considered. Additionally, for each four digit postal code district, population density per km^2^, all-cause mortality and proportion of persons with an alcohol-related disorder were considered in regression analysis on aggregated data.

Alcohol-related disorder was defined as one inpatient F10 diagnosis without F10.0 (i. e. acute intoxication) or three F10 prescriptions within four quarters of a year in the outpatient sector and was used to adjust the models to correct for its influence on cancer incidence [47, 48].

### Statistical analysis

All the analyses were conducted with the program R, version 3.3.2 (Vienna, Austria) R Core Team [49], but data preprocessing was done with the software Stata (StataCorp. 2013. *Stata Statistical Software: Release 13*. College Station, TX: StataCorp LP). The individual associations between residential air pollutants, green space and cancer incidence rates were assessed by multilevel Poisson models with 95% confidence intervals with the R software package *lme4*. Due to the fact that not all individuals were fully insured or alive in the five year-long study period (2010–2014), we used the exact observation time in days as offset in the models. Because of high correlations (Pearson correlations above |0.75|) between PM_10_, NO_2_, and NDVI, associations with each exposure were analyzed individually (see Additional file 5: Figure S1).

For the aggregated data, cancer incidence rates of the 186 four digit postal codes were age-standardized by the European standard population [50] and adjusted for measurement errors due to the limited observation period using conditional autoregressive models (CAR), which use the first order spatial dependencies (shared borders) with the software *BayesX* for R [51, 52]. These associations were analyzed with linear models.

We used the best subset approach according to the lowest Akaike Information Criterion (AIC) for variable selection, implemented in the R package *glmulti* [53], to filter out unassociated variables for each model (adjusted models). We computed crude and adjusted models for each outcome and exposure pair (Additional file 6: Table S5).

We conducted a sensitivity analysis for the influence of the change of residence on the effect estimates by exclusion of movers and by comparing these results with the primary analysis where both, movers and non-movers, were included (Additional file 7: Table S4).

## Results

### Baseline characteristics of the study population

Of more than 1.9 million initially cancer-free persons included into the analysis (Table 1), 3107 people developed mouth and throat cancer, 33,178 NMSC, 9611 prostate cancer, 9577 breast cancer, and 11,975 colorectal cancer during the follow-up period (2010–2014). Although there were slightly more females in the total population (53%), more males were affected by mouth and throat (74 vs. 26%) and colorectal cancer (53 vs. 47%), respectively. The number of incident cancer cases increased with age as expected, but decreased for the oldest age-groups (80–89, 90+). Mean age for developing cancer of interest ranged from 65 to 76 years. In the mouth and throat cancer subgroup, the proportion of people with an alcohol-related disorder reached almost 36%. Altogether, 69,722 people had a prevalent alcohol-related disorder (~ 3.6% from total) and 302,818 people (~ 15.8% from total) changed their place of residence between different four digit postal code districts at least once within the study period.

### Air pollution and green space

**Fig. 1:**
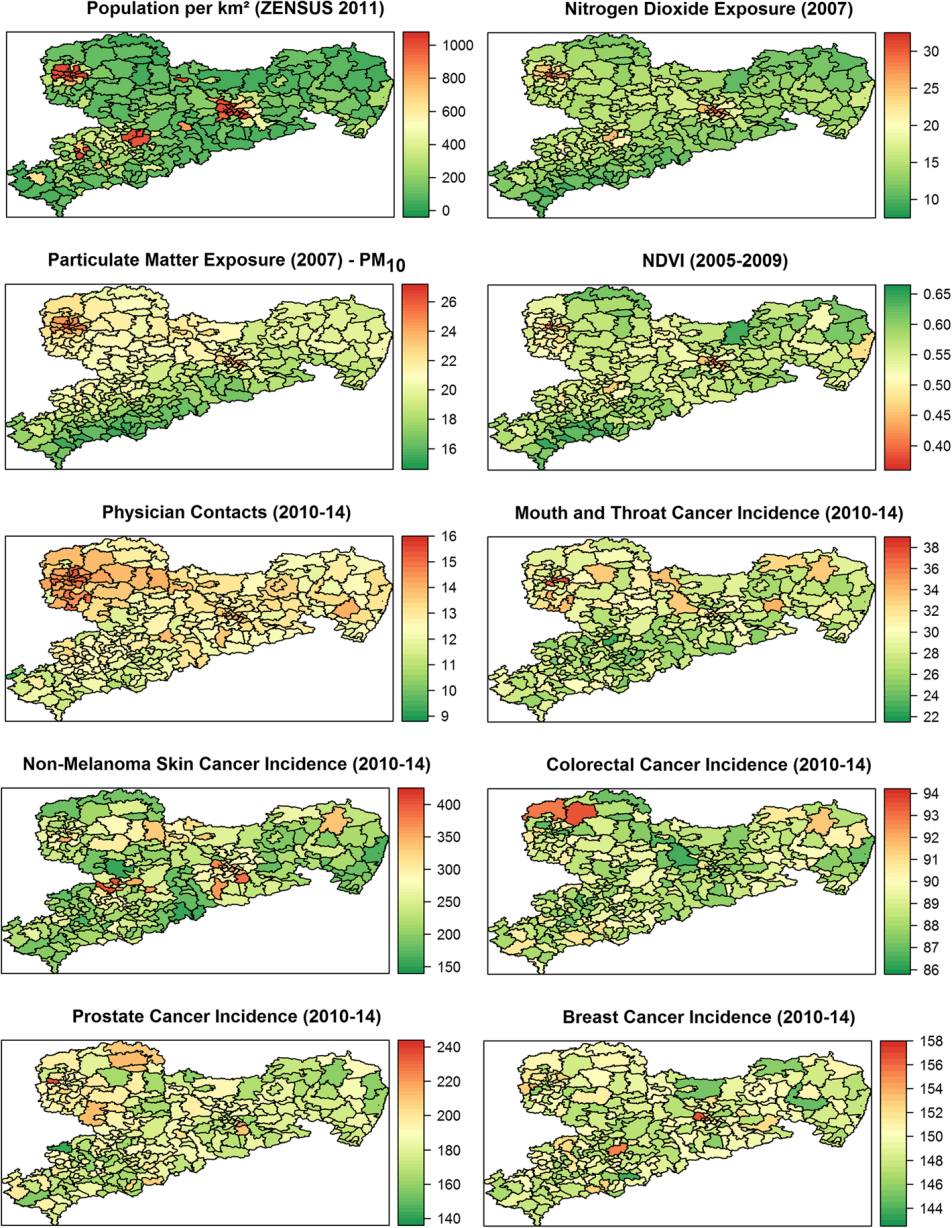
*Exposure and Case maps*; Mean concentrations of the exposures PM_10_ and NO_2_ (μg/m^3^) and mean NDVI (0 to + 1) are shown. Case maps of specific cancer types in Saxony, population density and mean physician contacts per year over the years 2010 until 2014 (data source: AOK PLUS) are given. Age-standardized cancer incidences (per 100.000 persons) were smoothed using a Bayesian CAR model

PM_10_ and NO_2_ levels were differentially distributed over the 186 four digit postal code districts in Saxony (Fig. 1). While NO_2_ showed highest concentrations in agglomeration areas with high population density (cities Dresden, Leipzig, Chemnitz, and Zwickau), PM_10_ was much smoother distributed, even in rural areas, with highest concentrations in the north-west part of Saxony around Leipzig and in Middle Saxony to Dresden. Lowest concentrations of PM_10_ were observed in the Erzgebirge region in the south-west of Saxony. On the contrary, green space, as defined by NDVI, was higher in rural areas and the lowest in the metropolitan areas of Leipzig, Dresden, Chemnitz, and Görlitz and their vicinities. PM_10_ concentrations ranged from 15.47 to 26.30 μg/m^3^ (mean: 20.89), NO_2_ concentrations ranged from 9.32 to 31.55 μg/m^3^ (mean: 20.44), and NDVI varied between 0.38 and 0.64 (mean: 0.51). The admissible annual averages for PM_10_ and NO_2_ in accordance with the Federal Immission Control Act (39. BImSchV) and the European Directive on Air Quality (2008/50/EG) are 40 μg/m^3^ each.

Additional file 5: Figure S1 shows Pearson correlation coefficients for associations between AP and NDVI, as well as physician contacts (PC). Most correlations were at least 75%, except for NDVI and PC (~ − 58%) or NO_2_ and PC (~ 61%).

### Association between air pollution, green space, and cancer incidence

#### Semi-individual data modeling

An increase in PM_10_ of 10 μg/m^3^ was associated with a 53% increase in relative risk (RR) of mouth and throat cancer and a 52% increase in RR of NMSC (Table 2). Prostate and breast cancer were modestly associated with PM_10_ with an increase in RR of 23 and 19%, respectively. The associations with N0_2_ were in the same direction as PM_10_ but the effect estimates were much weaker (7–24%) and did not reach statistical significance for prostate cancer. An increase in NDVI by 10% revealed associations with mouth and throat cancer with an 11% decrease in RR and with NMSC with a 16% decrease in RR. No associations were found for prostate or breast cancer and NDVI. Colorectal cancer was not affected by any of the exposures (Table 2).

**Table 2:** Relative risk (RR) estimates from multilevel Poisson regression models with observation time as offset and controlled for age as cubic term; 1.9 Mio. Persons in 186 postal code districts in Saxony were considered. 95% Wald confidence intervals (CI) are given in brackets; NMSC – non-melanoma skin cancer

	Colorectal cancer	Mouth and throat cancer	NMSC	Prostate cancer	Breast cancer
	RR (95% CI)	RR (95% CI)	RR (95% CI)	RR (95% CI)	RR (95% CI)
PM_10_ (per 10 μg/m^3^)	0.95 (0.87–1.04)	1.53 (1.31–1.78)	1.52 (1.35–1.72)	1.23 (1.08–1.39)	1.19 (1.09–1.31)
Male sex	1.78 (1.71–1.84)	2.70 (2.48–2.94)	1.61 (1.57–1.64)	/	/
Alcohol-related disorder	1.50 (1.38–1.63)	9.32 (8.62–10.07)	/	0.98 (0.90–1.07)	1.22 (1.06–1.41)
N0_2_ (per 10 μg/m^3^)	0.96 (0.92–1.00)	1.10 (1.01–1.19)	1.24 (1.16–1.32)	1.06 (0.99–1.12)	1.07 (1.03–1.12)
Male sex	1.78 (1.71–1.84)	2.70 (2.48–2.93)	1.61 (1.57–1.64)	/	/
Alcohol-related disorder	1.50 (1.38–1.63)	9.36 (8.66–10.12)	/	0.99 (0.90–1.08)	1.22 (1.06–1.41)
NDVI (per 10%)	1.03 (0.98–1.07)	0.89 (0.83–0.96)	0.84 (0.79–0.90)	0.95 (0.90–1.01)	0.96 (0.92–0.99)
Male sex	1.78 (1.71–1.84)	2.70 (2.48–2.93)	1.61 (1.57–1.64)	/	/
Alcohol-related disorder	1.50 (1.38–1.63)	9.35(8.65–10.11)	/	0.99 (0.90–1.08)	1.22 (1.06–1.41)

Alcohol-related disorder was associated with a more than 9 times increased risk of mouth and throat cancer a 50% increase of colorectal cancer and a 22% increase of breast cancer. Men were also more affected by any of the cancers which occur in both sexes (RR 1.6 to 2.7×; Table 2).

When only the people who did not change the place of residence were considered, similar results were observed except for the effect of PM_10_ on mouth and throat cancer which increased by 8% (Additional file 7: Table S4).

#### Aggregated data analysis

Cancer incidence rates generally showed a scattered distribution over Saxony (Fig. 1). The range of differences between the four digit postal code districts was small for colorectal cancer (86 to 94 cases per 100.000 insured persons) and breast cancer (144 to 158 cases per 100.000 insured persons), but high for mouth and throat cancer (22 to 36 cases), prostate cancer (140 to 240 cases), and especially for NMSC (150 to 400 cases). High correlation of physician contacts with PM_10_ (~ 75%) prevented using this variable of personal demand as a confounder in the adjusted models.

**Fig. 2:**
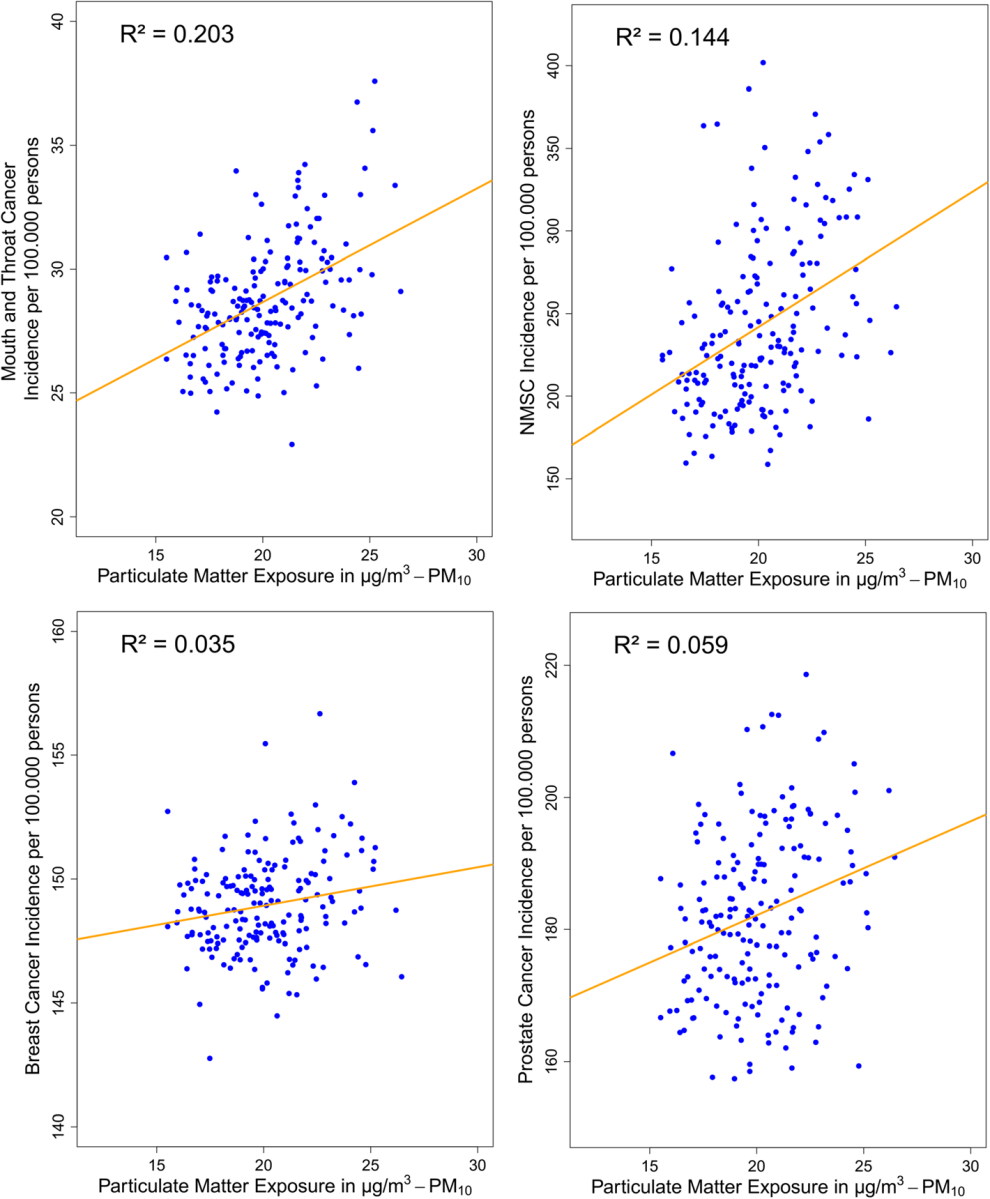
*Scatter plots of the crude linear regression analysis*; Assoziations between PM_10_ in μg/m^3^ and age-standardized cancer incidence rates per 100.000 persons. For each diagram, coefficients of determination (R^2^) are given which correspond to a moderate model fit for mouth and throat cancer and for NMSC, but show only a poor model fit for breast- and prostate cancer

Crude linear regression analyses showed associations between PM_10_ and mouth and throat cancer (R^2^ 0.203) or NMSC (R^2^ 0.144). Associations with breast- or prostate cancer were weak (Fig. 2). NO_2_ was associated with NMSC (R^2^ 0.164), but asssociations with breast-, and prostate cancer, or colorectal cancer were weak (Additional file 8: Figure S2 and Additional file 6: Table S5 in supplement). Moderate negative correlations were observed for NDVI and mouth and throat cancer (R^2^ 0.114) or NMSC (R^2^ 0.137), but again, the association with prostate cancer was weak (Additional file 8: Figure S2 and Additional file 6: Table S5).

Adjusted linear regression models were in the same direction as the crude analysis. Also PM_10_ and mouth and throat cancer (R^2^ 0.331) or NMSC (R^2^ 0.258) showed associations, but effect estimates were lower compared to the crude analyses (Additional file 6: Table S5). NO_2_showed also some association with NMSC (R^2^ 0.259) in the adjusted analysis, while associations to prostate-, breast-, and colorectal cancer remained weak. Negative correlations between NDVI and mouth and throat cancer (R^2^ 0.275) or NMSC (R^2^ 0.250) were still present in the adjusted analysis, but again, effect estimates were lower than in the crude models.

## Discussion

To the best of our knowledge, this study is novel and substantially adds to the current knowledge, because first of all the modeling is based on a very large cohort (1.9 million beneficiaries), which is needed to study associations between environmental exposures and cancer incidence. While earlier work has exclusively focused on the association between AP and lung cancer [1, 12–19, 54–56], we observed that PM_10_ is also related to mouth and throat cancer, NMSC, and maybe also to prostate and breast cancer. It is not so surprising that other types of cancer such as mouth and throat cancer or NMSC are also associated with AP, since chemical compounds are inhaled from the tidal air or even passed through the skin into the human body and accumulate there in the respective tissue over time. Some of these compounds could induce different forms of DNA damage [20–22], which could lead to tumor development. On the other hand, no association was found with colorectal cancer. Perhaps the carcinogens of the ambient air cannot accumulate in the intestine as they are already stopped by the lung or skin tissue beforehand. Associations between cancer incidence and green space (NDVI) or NO_2_ were present, but much smaller than for PM_10_.

We speculate that the high regional variation in NMSC (Fig. 1) could be due to differences in health care utilization in the population, whereas this is unlikely for more severe cancer types. One Australian study reported a higher skin cancer risk for regions with more vegetation [27], but exposure to UV light is certainly significantly greater in Australia compared to our study setting. Our results indicate that green space might be protective against NMSC in Germany. This observed protective effect might be secondary as the distribution of green spaces and PM_10_ were highly inversely correlated, and, as aforementioned, there are fewer emission sites around green spaces [31]. Thus, in areas where the UV intensity is much weaker than in Australia like in Germany, residential green space could lower the skin cancer risk. However, more studies are needed to clarify whether residing in greener places is beneficial or detrimental for NMSC and if this effect is causal or only indirectly transmitted through a lack of AP at these sites.

In the age group 18 until 64 years 5.0% of the sample population had an alcohol-related disorder. Survey data from 2012 for Germany and this age group found 3.1% with alcohol abuse and additionally 3.4% with alcohol dependency according to DSM IV. Our 5.0% estimate lies between 3.1 and 6.5% [57]. We expect therefore, that not all cases of alcohol abuse or dependency have already been recognized by physicians, but yet we consider it a valuable confounding variable. Further, a recent meta-analysis [58] and other studies [48, 59] found positive associations with high alcohol consumption on colorectal, mouth and throat, as well as breast cancer, and limited evidence for prostate cancer. Our effect estimate of alcohol on mouth and throat cancer is possibly a bit overestimated, as part of the people with an alcohol-related disorder are also smokers and these risk factors interact with each other [59].

We speculate that the high correlation of physician contacts and PM_10_ (~ 75%) suggests that PM_10_ is a wide-ranging risk factor for many diseases and therefore reducing it should be a central focus for community health.

### Strength and limitations

One of the strengths of the presented analysis is that the dataset contains a huge population. The population covers almost half of the residential population of Saxony and is regarding age and sex distribution very similar to the general population of Saxony, which allows us to investigate cancer incidence for the federal state of Saxony. Further, the study offers complete information about out- and inpatient treatments and is not affected by sampling or nonresponse bias. Selection bias because of changes of the health insurance due to the cancer disease is also unlikely, since no differences in cancer treatment between statutory health insurances are present in Germany. We have invested a lot of work in exact case definitions and can therefore build our analyses on valid cancer diagnoses.

Nevertheless, several important limitations should be acknowledged. Address information up to four digit postal code districts was available, which did not allow a high spatial resolution of individual outcome and exposure assessments. Therefore, we could only use the exposure of the four digit postal code district as a surrogate for the individual exposure (semi-individual). Additional information on some potentially important individual confounders, such as smoking, socioeconomic status, diet, or physical activity was unfortunately not available, which could have led to residual confounding. Further confounding could occur due to genetic predisposition of patients for certain cancer types, or due to other environmental pollutants, like pesticides. But farm workers, with a potential high exposition with pesticides, are not included in our data set, since they have their own statutory health insurance in Germany. Unfortunately, we had no information about occupation, indoor radon exposure of patients and virus infections possible related to cancer which could also confound our analyses.

The observed associations could therefore be overestimated or be biased due to exposure misclassification, for example, but the magnitude should not be too high. In one study on lung cancer, the hazard ratio of PM_10_ decreased by only 11% if it was additionally controlled for smoking status, smoking intensity, square of smoking intensity, smoking duration, time since quitting smoking, environmental tobacco smoke, occupation, fruit intake, marital status, education level, and employment status [54]. Our models assumed that the residential population did not move and AP was constant over time. Both assumptions, especially the first, are unlikely. Nevertheless, it is reassuring that our findings remained robust, when analyzing only the population that did not move outside their four digit postal code district during the observation period. Still, it is unknown what their movement history was until 2007. From the literature it is known that migrants have better health because people with low socioeconomic status or severe diseases are less mobile [60]. This could bias our results and therefore, we trust our primary analysis more than the results of the sensitivity analysis with excluded migrants.

## Conclusions

Beyond the current study situation we found some evidence that higher ambient AP levels increase the risk of mouth and throat cancer and of NMSC, while a higher residential green space level might have a protective effect. In summary, we assume that our effect estimates are not strongly biased by residual confounding, but we cannot exclude that for sure. Further research should try to measure the environmental exposures through the life course and focus on the effects of relocation together with environmental factors and extent the analyses to other cancer types.

## Additional files

Additional file 1: **Figure S3**. STROBE diagram; Flowchart showing selection of incident cancer cases following the guidelines of the STROBE initiative (STrengthening the Reporting of OBservational studies in Epidemiology). (PNG 26412 kb)

Additional file 2: **Table S1**. ICD-10 Codes; Analyzed cancer entities and corresponding ICD-10-GM codes are shown. (DOCX 12 kb)

Additional file 3: **Table S2**. *Validation of outpatient cancer cases other than NMSC*; Used OPS-, EBM codes and prescribed medications in outpatient cancer care. (DOCX 14 kb)

Additional file 4: **Table S3.** *Validation of outpatient cancer cases (NMSC)*; Used OPS- and EBM codes in outpatient cancer care of NMSC. (DOCX 14 kb)

Additional file 5: **Figure S1.** *Correlation plot*; Correlation between AP (PM_10_; NO_2_), NDVI, and physician contacts. Numbers are Pearson correlation coefficients. (PNG 1050 kb)

Additional file 6: **Table S5.** *Crude and adjusted linear regression of aggregated data*; Results of the linear regression analysis are shown. Estimators, 95% confidence intervals (CI) and coefficients of determination (R^2^) are given for crude and adjusted models. (DOCX 19 kb)

Additional file 7: **Table S4.** *Non-mover multilevel Poisson regression (CIs are Wald confidence intervals)*; Relative risk estimates for an increase of environmental exposures (10 μg/m^3^ for PM_10_ / N0_2_; 10% for NDVI) on cancer incidence in Saxony for patients with no relocation within study period between 2010 until 2014. (DOCX 15 kb)

Additional file 8: **Figure S2.** *Scatter plots of the crude linear regression analysis*; Associations between PM_10_, NDVI, and NO_2_ and different cancer types. For each diagram, coefficients of determination (R^2^) are given. We see positive but weak associations between NO_2_ and mouth and throat cancer, prostate cancer and breast cancer, but an elevated positive association with NMSC exists (R^2^ = 0.164). With increasing vegetation level (NDVI) of the neighborhood, cancer incidence rates decrease for breast cancer, prostate cancer, mouth and throat cancer, and NMSC (in increasing order). (PNG 1295 kb)
**Supplemental introduction text**
All case definitions were based on the respective coding systems for diagnosis (ICD-10-GM), procedures (Uniform Value Scale (EBM) and German modification of the International Classification of Procedures in Medicine (OPS)), as well as prescriptions (Anatomical Therapeutic Chemical code - ATC and pharmaceutical registration numbers - PZN).

## Main text

AIC: Akaike information criterion
AP: Air pollution
DSM: Diagnostic and Statistical Manual of Mental Disorders
NDVI: Normalized Difference Vegetation Index
NMSC: Non-melanoma skin cancer
NO_2_: Nitrogen dioxide
PC: Physician contacts
PM: Particulate matter
RR: Relative risk

## Supplement

EBM: Uniform Value Scale
OPS: German modification of the International Classification of Procedures in Medicine
PZN: Pharmaceutical registration numbers
