# Supplementary figures and images for: Outdoor air pollution, green space, and cancer incidence in Saxony: a semi-individual cohort study
Source: BMC Public Health. 2018 Jun 8;18:715. doi: 10.1186/s12889-018-5615-2 (PMC5994126; doi:10.1186/s12889-018-5615-2)

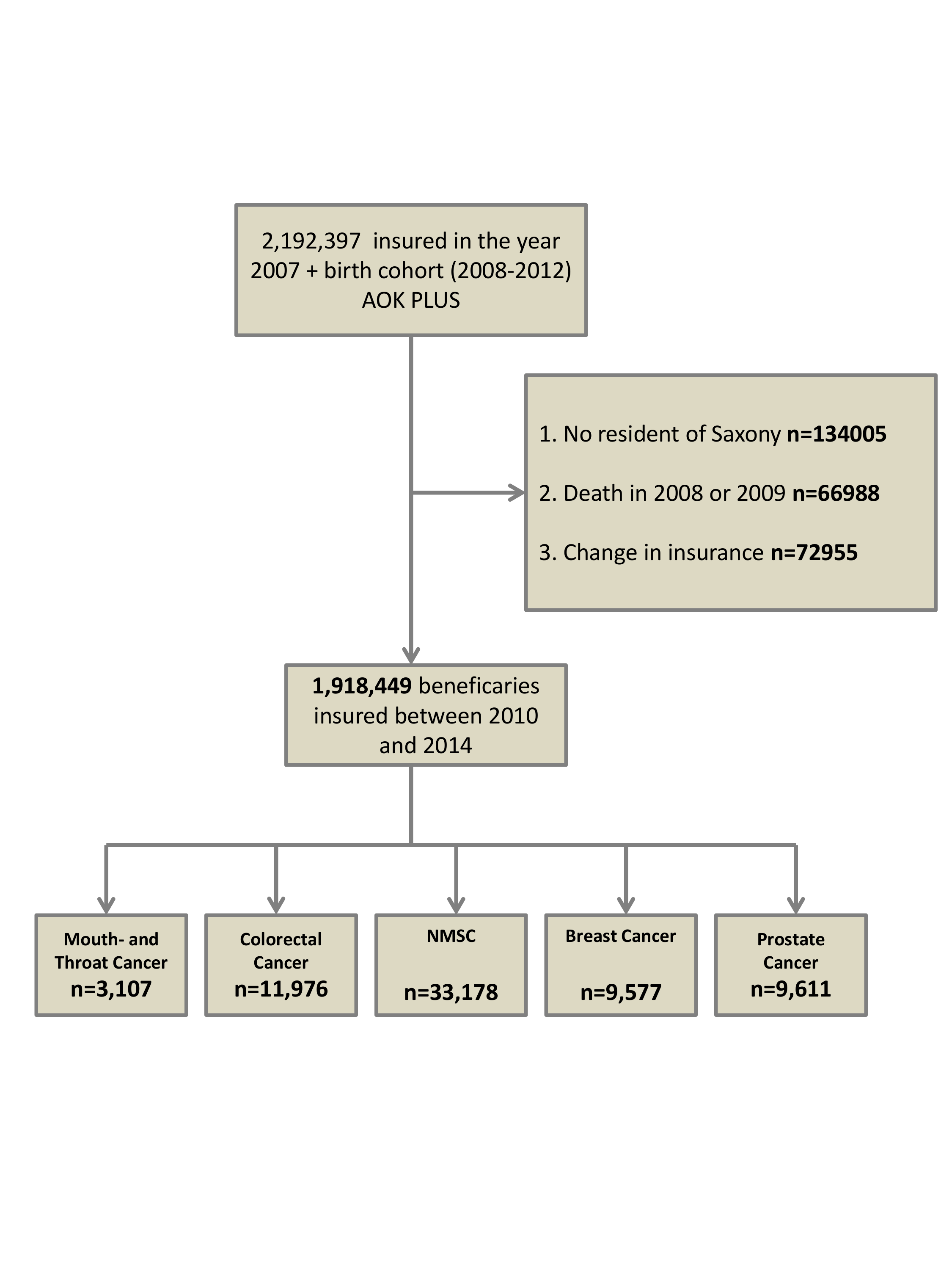

Supplement: Supplementary file 1 — Figure S3. STROBE diagram; Flowchart showing selection of incident cancer cases following the guidelines of the STROBE initiative (STrengthening the Reporting of OBservational studies in Epidemiology). (PNG 26412 kb) [file 12889_2018_5615_MOESM1_ESM.png]

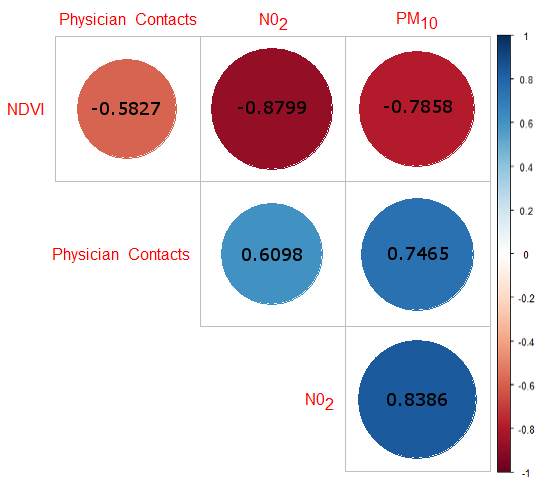

Supplement: Supplementary file 5 — Figure S1. Correlation plot; Correlation between AP (PM10; NO2), NDVI, and physician contacts. Numbers are Pearson correlation coefficients. (PNG 1050 kb) [file 12889_2018_5615_MOESM5_ESM.png]

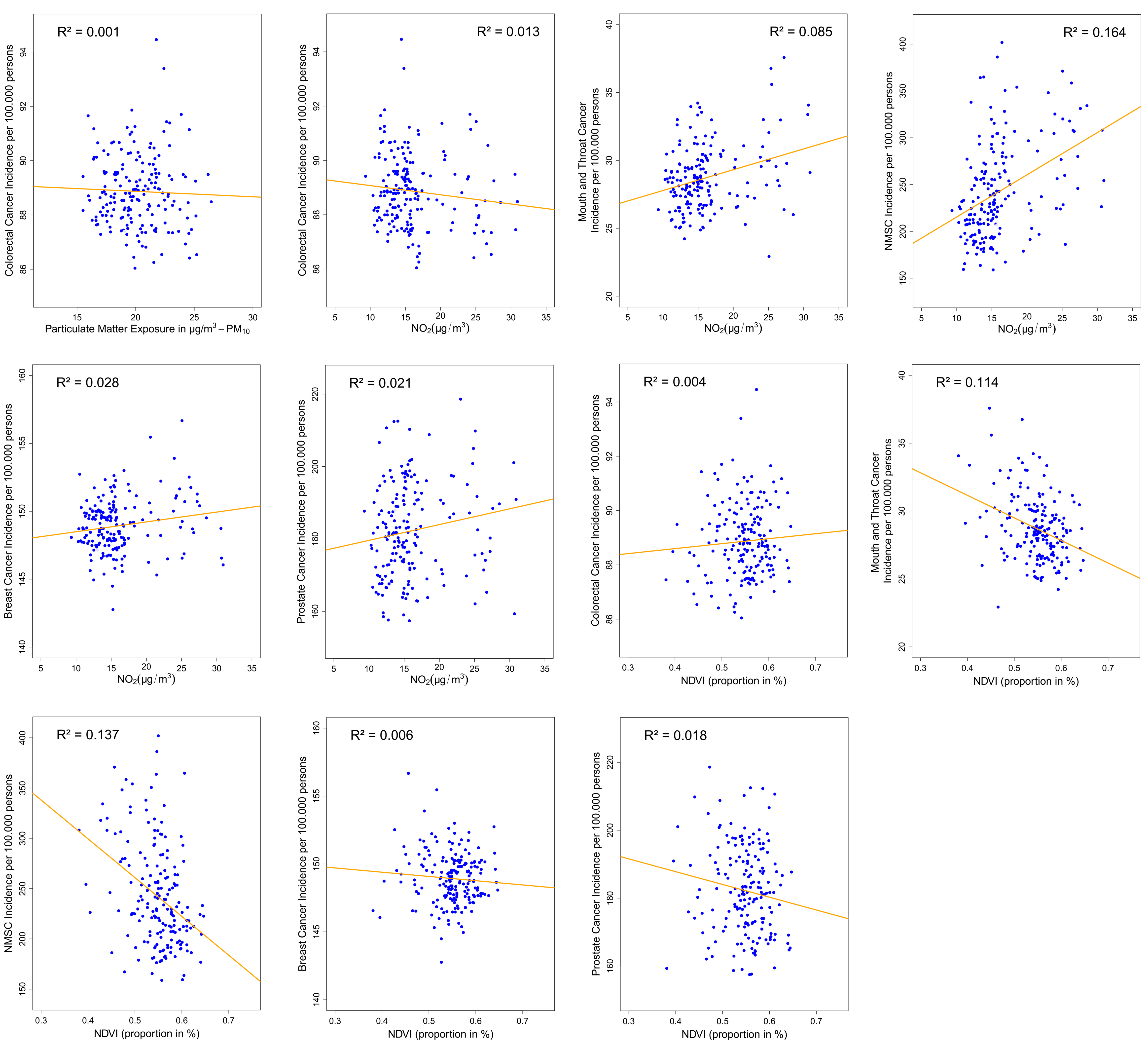

Supplement: Supplementary file 8 — Figure S2. Scatter plots of the crude linear regression analysis; Associations between PM10, NDVI, and NO2 and different cancer types. For each diagram, coefficients of determination (R2) are given. We see positive but weak associations between NO2 and mouth and throat cancer, prostate cancer and breast cancer, but an elevated positive association with NMSC exists (R2 = 0.164). With increasing vegetation level (NDVI) of the neighborhood, cancer incidence rates decrease for breast cancer, prostate cancer, mouth and throat cancer, and NMSC (in increasing order). (PNG 1295 kb) Supplemental introduction text All case definitions were based on the respective coding systems for diagnosis (ICD-10-GM), procedures (Uniform Value Scale (EBM) and German modification of the International Classification of Procedures in Medicine (OPS)), as well as prescriptions (Anatomical Therapeutic Chemical code - ATC and pharmaceutical registration numbers - PZN). [file 12889_2018_5615_MOESM8_ESM.png]
