# Supplementary material for: Outdoor air pollution, green space, and cancer incidence in Saxony: a semi-individual cohort study
Source: BMC Public Health. 2018 Jun 8;18:715. doi: 10.1186/s12889-018-5615-2 (PMC5994126; doi:10.1186/s12889-018-5615-2)
Supplement: Supplementary file 2 — Table S1. ICD-10 Codes; Analyzed cancer entities and corresponding ICD-10-GM codes are shown. (DOCX 12 kb) [file 12889_2018_5615_MOESM2_ESM.docx]

| Cancer types | ICD-10-GM Codes |
| --- | --- |
| Colorectal cancer | C18-C21 |
| Mouth and throat cancer | C00-C14 |
| Prostate cancer | C61 |
| Breast cancer (only women) | C50 |
| Non-melanoma skin cancer | C44 or L57.0 |
