# Supplementary material for: Outdoor air pollution, green space, and cancer incidence in Saxony: a semi-individual cohort study
Source: BMC Public Health. 2018 Jun 8;18:715. doi: 10.1186/s12889-018-5615-2 (PMC5994126; doi:10.1186/s12889-018-5615-2)
Supplement: Supplementary file 3 — Table S2. Validation of outpatient cancer cases other than NMSC; Used OPS-, EBM codes and prescribed medications in outpatient cancer care. (DOCX 14 kb) [file 12889_2018_5615_MOESM3_ESM.docx]

| German outpatient treatment documentation | Codes |
| --- | --- |
| OPS | 8-52 to 8-54 |
| EBM radiation | 25211, 25214, 25310, 25320, 25321, 25322, 25323, 25340, 25341, 25342 |
| EBM oncological treatment | 07345, 08345, 09345, 10345, 13435, 13675, 15345, 26315 |
| Medication | Cytostatic (special PZN) |
