# Supplementary material for: Outdoor air pollution, green space, and cancer incidence in Saxony: a semi-individual cohort study
Source: BMC Public Health. 2018 Jun 8;18:715. doi: 10.1186/s12889-018-5615-2 (PMC5994126; doi:10.1186/s12889-018-5615-2)
Supplement: Supplementary file 4 — Table S3. Validation of outpatient cancer cases (NMSC); Used OPS- and EBM codes in outpatient cancer care of NMSC. (DOCX 14 kb) [file 12889_2018_5615_MOESM4_ESM.docx]

| German outpatient treatment documentation | Codes |
| --- | --- |
| EBM | 10341, 10342, 10343, 10344 |
| OPS | 5-091, 5-181, 5-182.1, 5-182.3, 5-212, 5-894, 5-895, 5-898.6, 5-913, 5-914, 5-915, 5-984, 5-985, 8-560.4 |
