# Supplementary material for: Outdoor air pollution, green space, and cancer incidence in Saxony: a semi-individual cohort study
Source: BMC Public Health. 2018 Jun 8;18:715. doi: 10.1186/s12889-018-5615-2 (PMC5994126; doi:10.1186/s12889-018-5615-2)
Supplement: Supplementary file 6 — Table S5. Crude and adjusted linear regression of aggregated data; Results of the linear regression analysis are shown. Estimators, 95% confidence intervals (CI) and coefficients of determination (R2) are given for crude and adjusted models. (DOCX 19 kb) [file 12889_2018_5615_MOESM6_ESM.docx]

| **Carcinogen: PM10** |  |  |  |  |  |  |
| --- | --- | --- | --- | --- | --- | --- |
| **Outcome: prostate cancer incidence** | | | **crude** |  | **adjusted** |  |
| *predictors* | *estimate* | *95% CI* | *R²* | *estimate* | *95% CI* | *R²* |
|  |  |  |  |  |  |  |
| PM10 | 1.426 | 0.599; 2.252 | 0.059 | 1.985 | 1.057; 2.912 | 0.090 |
| F10 diagnosis | <-0.001 | -0.003; 0.002 | <0.001 | -0.004 | -0.006; <-0.001 |  |
| **Outcome: breast cancer incidence** | |  |  |  |  |  |
|  |  |  |  |  |  |  |
| PM10 | 0.156 | 0.037; 0.274 | 0.035 | 0.156 | 0.037; 0.274 | 0.035 |
| **Outcome: NMSC incidence** | | |  |  |  |  |
|  |  |  |  |  |  |  |
| PM10 | 8.190 | 5.289; 11.09 | 0.144 | 5.289 | 2.375; 8.203 | 0.258 |
| mortality | -0.454 | -0.583; -0.324 | 0.206 | -0.363 | -0.498; -0228 |  |
| **Outcome: mouth and throat cancer incidence** | | |  |  |  |  |
|  |  |  |  |  |  |  |
| PM10 | 0.458 | 0.326; 0.59 | 0.203 | 0.351 | 0.187; 0.515 | 0.331 |
| F10 diagnosis | 0.002 | 0.001; 0.002 | 0.265 | 0.001 | <0.001; 0.002 |  |
| population/km² | <0.001 | <0.001; 0.0008 | 0.050 | <-0.001 | < -0.001; -0.00003 |  |
| **Carcinogen: NO2** |  |  |  |  |  |  |
| **Outcome: prostate cancer incidence** | | | **crude** |  | **adjusted** |  |
|  |  |  |  |  |  |  |
| NO2 | 0.436 | <0.001; 0.872 | 0.021 | 0.631 | 0.139; 1.123 | 0.035 |
| F10 diagnosis | <-0.001 | -0.003; 0.002 | 0.001 | -0.002 | -0.005; 0.0005 |  |
| **Outcome: breast cancer incidence** | |  |  |  |  |  |
|  |  |  |  |  |  |  |
| NO2 | 0.073 | 0.011; 0.134 | 0.028 | 0.073 | 0.011; 0.134 | 0.028 |
| **Outcome: NMSC incidence** | | |  |  |  |  |
|  |  |  |  |  |  |  |
| NO2 | 4.51 | 3.029; 5.99 | 0.164 | 2.837 | 1.281; 4.393 | 0.259 |
| mortality | -0.454 | -0.583; -0.324 | 0.206 | -0.342 | -0.482; -0.202 |  |
| **Outcome: colorectal cancer incidence** | | |  |  |  |  |
|  |  |  |  |  |  |  |
| NO2 | -0.034 | -0.076; 0.009 | 0.013 | -0.052 | -0.105; -0.0002 | 0.099 |
| F10 diagnosis | <0.001 | <0.001; 0.0006 | 0.031 | <0.001 | 0.0002; 0.0007 |  |
| mortality | 0.005 | 0.002; 0.009 | 0.043 | 0.004 | <-0.001; 0.008 |  |
| **Possible protection factor: NDVI/Green space** | | |  |  |  |  |
| **Outcome: prostate cancer incidence** | | | **crude** |  | **adjusted** |  |
|  |  |  |  |  |  |  |
| NDVI/green space | -37.700 | -78.50; 3.185 | 0.018 | -56.322 | -102.90;-9.743 | 0.032 |
| F10 diagnosis | <-0.001 | -0.003; 0.002 | 0.001 | -0.002 | -0.005;-0.0005 |  |
| **Outcome: NMSC incidence** | | |  |  |  |  |
|  |  |  |  |  |  |  |
| NDVI/green space | -385.800 | -526.60; -245.00 | 0.137 | -236.275 | -379.461; -93.090 | 0.250 |
| mortality | -0.454 | -0.583; -0.324 | 0.206 | -0.365 | -0.502; -0.227 |  |
| **Outcome: mouth and throat cancer incidence** | | |  |  |  |  |
|  |  |  |  |  |  |  |
| NDVI/green space | -16.61 | -23.34; -9.887 | 0.114 | -5.611 | -12.60; 1.382 | 0.275 |
| F10 diagnosis | 0.002 | 0.001; 0.002 | 0.265 | 0.001 | <0.001; 0.002 |  |

NMSC – non-melanoma skin cancer
