# Supplementary material for: Outdoor air pollution, green space, and cancer incidence in Saxony: a semi-individual cohort study
Source: BMC Public Health. 2018 Jun 8;18:715. doi: 10.1186/s12889-018-5615-2 (PMC5994126; doi:10.1186/s12889-018-5615-2)
Supplement: Supplementary file 7 — Table S4. Non-mover multilevel Poisson regression (CIs are Wald confidence intervals); Relative risk estimates for an increase of environmental exposures (10 μg/m3 for PM10 / N02; 10% for NDVI) on cancer incidence in Saxony for patients with no relocation within study period between 2010 until 2014. (DOCX 15 kb) [file 12889_2018_5615_MOESM7_ESM.docx]

|  | colorectal cancer | mouth and throat cancer | NMSC | prostate cancer | breast cancer |
| --- | --- | --- | --- | --- | --- |
|  | RR (95% CI) | RR (95% CI) | RR (95% CI) | RR (95% CI) | RR (95% CI) |
| PM_10_(per10 µg/m³) | 0.93 (0.84-1.03) | 1.61 (1.37-1.89) | 1.54 (1.37-1.75) | 1.23 (1.09-1.40) | 1.18 (1.07-1.31) |
| Male sex | 1.80 (1.73-1.87) | 2.73 (2.50-2.98) | 1.61 (1.57-1.64) | / | / |
| Alcohol-related disorder | 1.46 (1.34-1.60) | 9.19 (8.46-9.99) | / | 0.99 (0.90-1.08) | 1.18 (1.01-1.38) |
|  | | | | | |
| N0_2_ (per 10 µg/m³) | 0.96 (0.91-1.01) | 1.13 (1.05-1.23) | 1.25 (1.17-1.34) | 1.06 (0.99-1.13) | 1.07 (1.02-1.12) |
| Male sex | 1.80 (1.73-1.87) | 2.73 (2.49-2.98) | 1.61 (1.57-1.64) | / | / |
| Alcohol-related disorder | 1.46 (1.34-1.60) | 9.22 (8.49-10.0) | / | 0.99 (0.90-1.08) | 1.18 (1.01-1.38) |
|  | | | | | |
| NDVI (per 10%) | 1.03 (0.98-1.07) | 0.88 (0.81-0.95) | 0.83 (0.78-0.89) | 0.95 (0.90-1.01) | 0.96 (0.92-1.01) |
| Male sex | 1.80 (1.73-1.87) | 2.73 (2.49-2.98) | 1.61 (1.57-1.64) | / | / |
| Alcohol-related disorder | 1.46 (1.34-1.60) | 9.22 (8.48-10.00) | / | 0.99 (0.90-1.08) | 1.18 (1.01-1.38) |

NMSC – non-melanoma skin cancer
